# *Mycoplasma pneumoniae* downregulates RECK to promote matrix metalloproteinase-9 secretion by bronchial epithelial cells

**DOI:** 10.1080/21505594.2022.2101746

**Published:** 2022-07-26

**Authors:** Lianmei Qin, Lu Liu, Yueping Wu, Yiwen Chen, Yueyue Wu, Haodang Luo, Yixuan Xi, Feichen Xiu, Jun Hu, Liesong Chen, Ning Wu, Jun He, Yanhua Zeng, Cuiming Zhu, Xiaoxing You

**Affiliations:** a Institute of Pathogenic Biology, Hengyang Medical School, Hunan Provincial Key Laboratory for Special Pathogens Prevention and Control, Hunan Province Cooperative Innovation Center for Molecular Target New Drug Study, University of South China, Hengyang, China; b Department of Blood Transfusion, Shenzhen Children’s Hospital, Shenzhen, China; c Department of Clinical Laboratory, The Affiliated Nanhua Hospital, Hengyang Medical School, University of South China, Hengyang, China; d Department of Cardiothoracic Surgery, The Second Affiliated Hospital, Hengyang Medical School, University of South China, Hengyang, China; e Department of Clinical Laboratory, Hengyang No.1 People’s Hospital, Hengyang, China

**Keywords:** *Mycoplasma pneumoniae*, matrix metalloproteinase-9, reversion-inducing cysteine-rich protein with Kazal motifs (RECK), bronchial epithelial cell

## Abstract

Airway epithelial cells function as both a physical barrier against harmful substances and pathogenic microorganisms and as an important participant in the innate immune system. Matrix metalloproteinase-9 (MMP-9) plays a crucial role in modulating inflammatory responses during respiratory infections. However, the signalling cascade that induces MMP-9 secretion from epithelial cells infected with *Mycoplasma pneumoniae* remains poorly understood. In this study, we investigated the mechanism of MMP-9 secretion in airway epithelial cells infected with *M. pneumoniae*. Our data clearly showed that *M. pneumoniae* induced the secretion of MMP-9 from bronchial epithelial cells and upregulated its enzymatic activity in a time- and dose-dependent manner. Using specific inhibitors and chromatin co-precipitation experiments, we confirmed that the expression of MMP-9 is reliant on the activation of the Toll-like receptor 2 (TLR2) and TLR6-dependent mitogen-activated protein kinase/nuclear factor- κB/activator protein-1 (MAPK/NF-κB/AP-1) pathways. Additionally, epigenetic modifications such as histone acetylation and the nuclear transcription factor Sp1 also regulate MMP-9 expression. *M. pneumoniae* infection also decreased the expression of the tumour suppressor reversion-inducing cysteine-rich protein with Kazal motifs (RECK) by inducing Sp1 phosphorylation. Overexpression of RECK significantly impaired the *M. pneumoniae*-triggered increase in MMP-9 enzymatic activity, although the level of MMP-9 protein remained constant. The study demonstrated that *M. pneumoniae*-triggered MMP-9 expression is modulated by TLR2 and 6, the MAPK/NF-κB/AP-1 signalling cascade, and histone acetylation, and *M. pneumoniae* downregulated the expression of RECK, thereby increasing MMP-9 activity to modulate the inflammatory response, which could play a role in airway remodelling.

## Introduction

*Mycoplasma pneumoniae* is the smallest prokaryotic microorganism and causes community-acquired pneumonia (CAP). It can infect humans of any age but is more common in school-age children and adolescents [1]. In addition to respiratory infections, *M. pneumoniae* can cause extrapulmonary complications including myocarditis, pericarditis, nephritis, and meningitis [2,3]. Several factors contribute to the pathogenesis of *M. pneumoniae*, and activation of the host immune response has been proposed as a key factor in these complications [4,5]. Thus, it is critical to investigate the interactions between *M. pneumoniae* and host cells to better understand the etiopathogenesis of this disease.

The respiratory tract is the primary site of *M. pneumoniae* infections. Upon infection, *M. pneumoniae* attaches to bronchial epithelial cells via surface-exposed adhesins, which trigger a series of cascade reactions involving interactions with toll-like receptors (TLR1, TLR2, and TLR6) [6], mitogen-activated protein kinase (MAPK), nuclear factor-kappa B (NF-κB), and activated protein-1 (AP-1), leading to the production of diverse pro-inflammatory cytokines and inflammatory mediators [7–9]. The key steps in triggering the immune response involve leukocytes breaking through the basement membrane of blood vessels and degrading the extracellular matrix (ECM) to reach the site of infection [10,11]. Matrix metalloproteinase (MMP) activation is a prerequisite for ECM degradation [12]. MMPs belong to a family of Zn^2+^-dependent proteolytic enzymes, and more than 20 types of MMPs have been identified in humans. MMP-9 is an important indicator of lung inflammation [13].

MMP-9, also known as gelatinase B, is a secreted protease that specifically degrades type IV collagen in the ECM [14]. In addition, it can influence the host immune response through a variety of mechanisms, such as regulating the activities of certain chemokines like CXCL5 and CXCL6 to modulate leukocyte exudation [15], inducing high-affinity IL-2 receptor expression in T cells [16], and accelerating the maturation of certain cytokines like IL-1β and TNF-α [17,18]. Elevated secretion of MMP-9 was detected in patients with asthma, acute respiratory distress syndrome (ARDS), and chronic obstructive pulmonary disease (COPD) [19,20]. Recently, a prospective study also indicated that *MMP-9* mRNA expression in peripheral blood mononuclear cells (PBMCs) and MMP-9 protein levels in plasma were increased among hospitalized patients in the acute phase of CAP caused by *M. pneumoniae*, and plasma MMP-9 levels were correlated with leukocyte count in peripheral circulation [21]. However, the underlying mechanism remains unclear.

MMP-9 expression and activation are controlled at several levels, including transcription (NF-κB and AP-1), secretion, and enzymatic activity [22]. Recently, the tumour suppressor reversion-inducing cysteine-rich protein with Kazal motifs (RECK) was reported to function as a natural inhibitor of MMP-9 that not only prevents the release of MMP-9 from the membrane, but also affects its enzymatic activity, thus inhibiting tumour cell invasion and metastasis [23]. In patients with rheumatoid arthritis, the reduced expression of RECK in inflamed synovial membranes contributes to synovial proliferation and cartilage destruction [24]. Also, RECK plays a negative role in angiotensin II-induced cardiac fibrosis [25] by inhibiting MMP-9 activity. Therefore, RECK may not only be a tumour suppressor but also play a role in inflammatory diseases. However, whether RECK is involved in the regulation of MMP-9 secretion from epithelial cells infected with *M. pneumoniae* remains unclear.

Most studies on *M. pneumoniae* and the associated inflammatory responses have mainly focused on monocytes and macrophages, while the role of airway epithelial cells has been largely ignored. In this study, we found that *M. pneumoniae* infection could induce MMP-9 secretion via multiple mechanisms, including activation of the MAPK/NF-κB/AP-1 pathway and promotion of histone acetylation in bronchial epithelial cells. In addition, *M. pneumoniae* infection activates nuclear transcription factor Sp1, which directly upregulates the transcription of MMP-9 and downregulates the expression of RECK. The reduced expression of RECK leads to a weakened inhibitory effect on MMP-9 enzyme activity and an increased secretion of mature MMP-9, which eventually causes ECM degradation, leukocyte exudation, and/or destruction of the epithelium, and may participate in other pathological reactions such as airway remodelling.

## Materials and methods

### Reagents and antibodies

The Mycoplasma Broth Base (CM1166) and Mycoplasma Supplement G (SR0059) were purchased from Oxoid (Basingstoke, Hampshire, UK). Bronchial epithelial cell medium (BEGM) BulletKit (CC-3171 and CC-4175) was purchased from Lonza (Walkersville, MD, USA). Anti-TLR1, anti-TLR2, and anti-TLR6 neutralizing antibodies and dominant negative (DN) plasmids DN-TLR1, DN-TLR2, and DN-TLR6 were purchased from InvivoGen (San Diego, CA). Antibodies against MMP-9, MAP kinase, p65, c-jun, c-fos, histone H3 and H4, histone deacetylase (HDAC) 1 and HDAC2, RECK, Sp1, and horse radish peroxidase (HRP)-conjugated secondary antibodies were purchased from CST (Danvers, MA, USA). Anti-phosphorylated Sp1 and anti-hTIMP-1 antibodies were obtained from Life (Gaithersburg, MD, USA) and NOVUS (Littleton, CO, USA), respectively. The MAP kinase inhibitors U0126, SP600125, and SB203580 were obtained from Abcam (Cambridge, UK) and BAY 11–7082 was obtained from CST. The HDAC inhibitor trichostatin A (TSA) and Sp1 inhibitor mithramycin A were purchased from Cayman (Ann Arbor, MI, USA).

### Bronchial epithelial cells culture

The bronchial epithelial cell line BEAS-2B was purchased from ATCC and maintained in BEGM culture without antibiotic supplements. Primary human bronchial epithelial (HBE) cells were isolated from tumour-free resected lung tissue from anonymous donors at the Department of Cardiothoracic Surgery with the approval of the Ethics Committee of the University of South China. Isolation and culture of HBE cells were performed as previously described [26]. To prepare the cell culture, the biopsies were washed with Earle’s Balanced Salt Solution, dissected from connective tissue, cut into pieces (2–3 mm^3^), and placed in collagen-coated 6-well plates to serve as a source of primary cells. The grown epithelial cells were transferred into collagen-coated 10-cm dishes as soon as they reached 70% confluence. The primary cell cultures were maintained at 37°C in a humidified incubator with 5% CO_2_. When confluent monolayers were observed, epithelial cells in the wells were infected with *M. pneumoniae*.

### M. pneumoniae culture and infection

*M. pneumoniae* strain 129 was obtained from ATCC (Manassas, VA) and grown in mycoplasma broth supplemented with 20% mycoplasma supplement G for approximately 5–7 days until the medium colour changed from red to orange. *M. pneumoniae* cells were harvested and passed through a 25-gauge needle ten times. A portion of the suspension was serially diluted and plated onto solid agar to determine colony-forming units (CFUs), as previously described [27]. For stimulation experiments, cells were seeded in 24- or 6-well plates and allowed to grow overnight before exposure to *M. pneumoniae* at various multiplicities of infection (MOIs) for the indicated time intervals.

### Quantitative real-time polymerase chain reaction (qPCR)

Total RNA was extracted using TRIzol reagent (Invitrogen) following the manufacturer’s instructions. Thereafter, cDNA was synthesized and amplified using ChamQ Universal SYBR qPCR Master Mix (Vazyme Biotech, China) in a LightCycler 96® instrument (Roche) in the following conditions: 95°C for 300 s, followed by 40 cycles of 95°C for 10 s and 59°C for 40 s. Relative target mRNA expression was normalized to *GAPDH* and calculated using the 2^−∆∆CT^ method [28]. The sequences of the primers used were as follows: MMP-9 (F) 5′-CAGTACCGAGAGAAAGCCTATT-3′ and (R) 5′-CAGGATGTCATAGGTCACGTAG-3′; RECK (F) 5′-GCACAACAATCTCTGCACTTTA-3′ and (R) 5′-CAGTCCCCATAGTAATCGACTG-3′; TIMP-1 (F) 5′-CATCACTACCTGCAGTTTTGTG-3′ and (R) 5′- TGGATAAACAGGGAAACACTGT-3′; GAPDH (F) 5- ACCACAGTCCATGCCATCAC-3′ and (R) 5′- TCCACCACCCTGTTGCTGTA-3′.

### Gelatin zymography

The cell culture supernatant, without reduction or thermal denaturation, was separated using 10% sodium dodecyl sulphate – polyacrylamide gel electrophoresis (SDS – PAGE) containing 1% gelatin. The gels were then incubated in washing buffer to remove SDS, rinsed with incubation buffer for 5–10 min, and incubated for 24–48 h at 37°C in a new buffer (1% Triton X-100, 50 mM Tris-HCl, 5 mM CaCl_2_, 1 μM ZnCl_2_ and 2% NaN_3_). The gels were stained with 0.5% Coomassie Brilliant Blue for 30–60 min and subsequently decolorized in a solution of 40% methanol and 10% acetic acid. Gelatinase activity appeared as a clear white band against a dark blue background, and the image was captured using a camera and an LED film lamp.

### HDAC activity assay

BEAS-2B cells were seeded at a density of 2 × 10^5^/ml. The cells were infected with *M. pneumoniae*, the supernatant discarded, and intracellular HDAC activity determined using an HDAC activity assay kit (Cayman) according to the manufacturer’s instructions. HDAC activity was calculated using the following formula: HDAC activity (nmol/min/ml) = [corrected sample fluorescence - (y-intercept)/slope] × dilution/15 min.

### Immunofluorescence staining for activation of NF-κB

BEAS-2B cells cultured in 6-well plates were fixed with paraformaldehyde and permeabilized with 0.3% Triton/phosphate-buffered saline, as previously described [29]. After permeabilization, cells were blocked with goat serum for 1 h at 37°C and incubated with a primary rabbit anti-p65 antibody overnight at 4°C. Next, the cells were washed and incubated with Alexa 488-conjugated secondary antibody (Abcam) in the dark at 37°C for 1 h. Nuclei were stained with 4′-6-diamidino-2-phenylindole (DAPI). Images were captured using an immunofluorescence microscope (Ts2 R, Nikon).

### Immunoblotting

The cells were lysed in radio immunoprecipitation assay lysis buffer (Pierce, Rockford, IL, USA) containing 1% protease and phosphatase inhibitors (Roche, Mannheim, Germany). Histones were extracted from BEAS-2B cells using a histone extraction kit (Abcam). Samples containing equal amounts of protein were separated by SDS – PAGE and transferred onto a poly(vinylidene fluoride) membrane (Millipore, Bedford, MA, USA). After blocking with 5% skimmed milk at 37°C for 1.5 h, the membranes were washed and incubated with the corresponding primary antibodies at 4°C overnight, and then incubated with HRP-linked secondary antibodies at 37°C for 1.5 h. Signals were visualized using SuperSignal™ West Dura Extended Duration Substrate (Pierce), and the bands were captured using the ECL system (G: BOX Chemi XX9, Syngene).

### Overexpression of dominant-negative TLRs and RECK

Transfection of the dominant-negative plasmids of TLR1, TLR2, TLR6, and RECK was accomplished using Lipofectamine3000 (Life Technologies, Carlsbad, CA, USA). A total of 4 × 10^5^ subcultured cells were transfected with 0.5 mg of DN-TLR1, DN-TLR2, DN-TLR6 or RECK plasmid (The pCXNneo-hRECK plasmid and the pCXNneo empty vector were kindly provided by Prof. Chiaki Takahashi, Cancer Research Institute, Kanazawa University, Japan). After 20 h of transfection, cells were infected with *M. pneumoniae* for further experiments.

### Chromatin immunoprecipitation

Chromatin immunoprecipitation (ChIP) was performed as described previously [30]. The obtained cell suspension was immersed in 1% formaldehyde for 10–15 min to crosslink the transcription factors to DNA, and chromatin from lysed nuclei was sheared into 200–600 bp fragments using a sonifier (VCX130; Sonics & Materials). For immunoprecipitation, 100 µg of sheared chromatin was incubated with 5 µg of an anti-c-jun primary antibody. Rabbit IgG antibody was used as the control. The primer pair used for the PCR was 5′-TGTCCCTTTACTGCCCTGA-3′ (F) and 5′-ACTCCAGGCTCTGTCCTCCTCTT-3′ (R).

### Statistical analysis

The obtained data were expressed as mean ± SEM and were analysed using one-way analysis of variance (ANOVA) followed by Tukey’s post hoc test. Differences with a *P* value <0.05 were considered statistically significant.

## Results

### M. pneumoniae induces the expression and secretion of MMP-9 in bronchial epithelial cells

**Figure 1.**
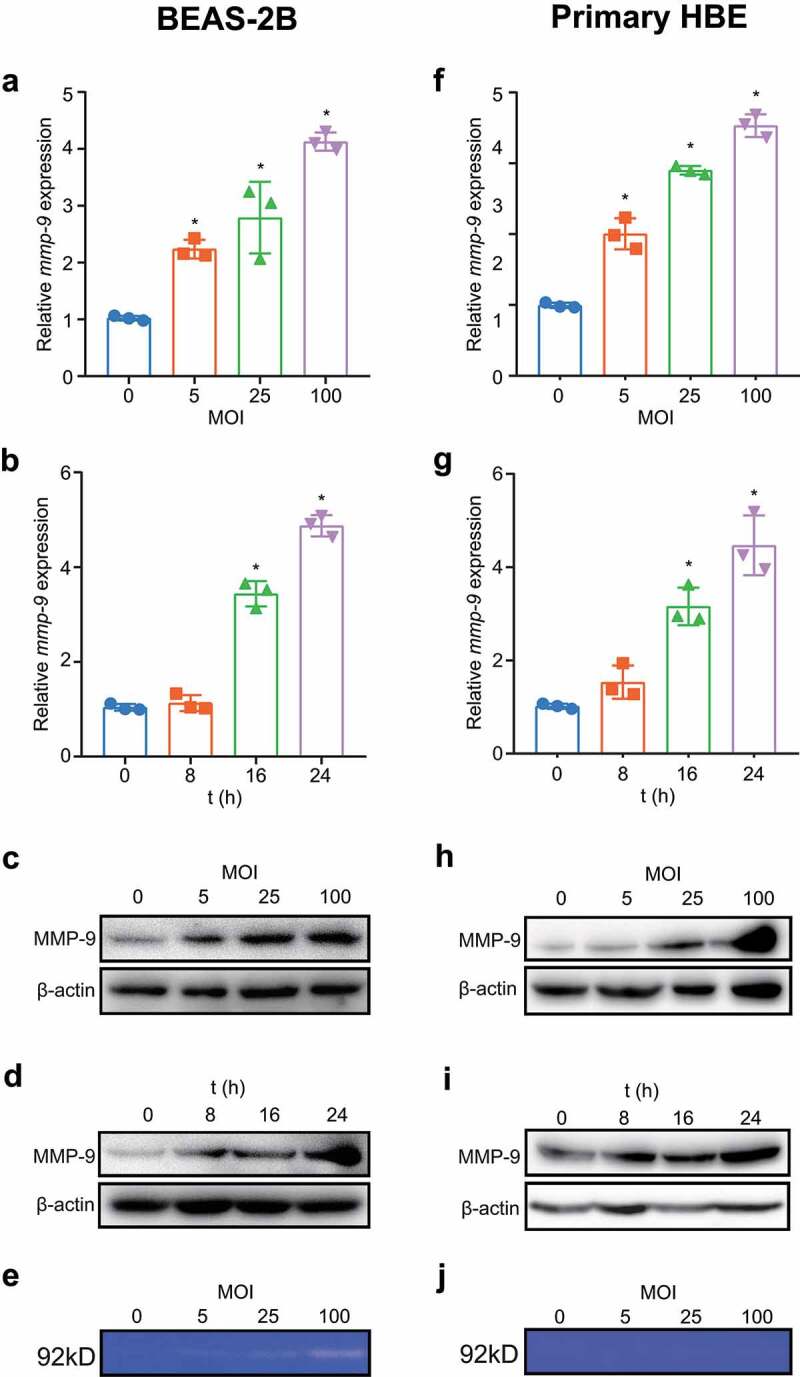
*M. pneumoniae* induces secretion of MMP-9 from bronchial epithelial cells. BEAS-2B cells or primary bronchial epithelial cells were infected with *M. pneumoniae* at a multiplicity of infection (MOI) of 0, 5, 25, and 100 for 16 h, or at an MOI of 100 for 8–24 h. The mRNA and protein expression levels were determined by qPCR and immunoblotting (a–d, f–i), and the enzymatic activity of MMP-9 in the supernatant was measured by gelatin zymography (e, j). Representative results from three independent experiments are shown. *P < 0.05, compared with the control group (0 MOI or 0 h).

It has been shown that patients with CAP caused by *M. pneumoniae* show increased expression of *MMP-9* in PBMCs as well as higher plasma concentrations of MMP-9 than healthy controls [21]. To clarify whether these events can also occur in vitro in bronchial epithelial cells, we first detected the inducible ability of *M. pneumoniae* both in the bronchial epithelial cell line BEAS-2B and in primary bronchial epithelial cells. Our results indicated that mRNA and protein levels of MMP-9 were upregulated in an MOI- and time-dependent manner after *M. pneumoniae* infection compared with the levels in uninfected control cells (Figure 1(a–I)). In vivo, MMP-9 is synthesized and secreted as an inactive zymogen that is activated by the removal of the propeptide by another protease [31]. Therefore, the culture supernatant was also analysed by gelatin zymography, and we found that the activity levels of secreted MMP-9 increased as the MOI increased (Figure 1(e–f)). Considering that *M. pneumoniae* infection induces TNF-α secretion [32], which has the ability to promote MMP-9 expression [33], we examined the possibility that MMP-9 production is mediated by endogenous TNF-α. However, pretreatment with a TNF-α-neutralizing antibody did not affect the induction of *MMP-9* mRNA by *M. pneumoniae* (data not shown). These results demonstrate that *M. pneumoniae* induces the expression and secretion of MMP-9 in bronchial epithelial cells.

### TLR2 and TLR6 are essential for MMP-9 expression upon M. pneumoniae infection

**Figure 2.**
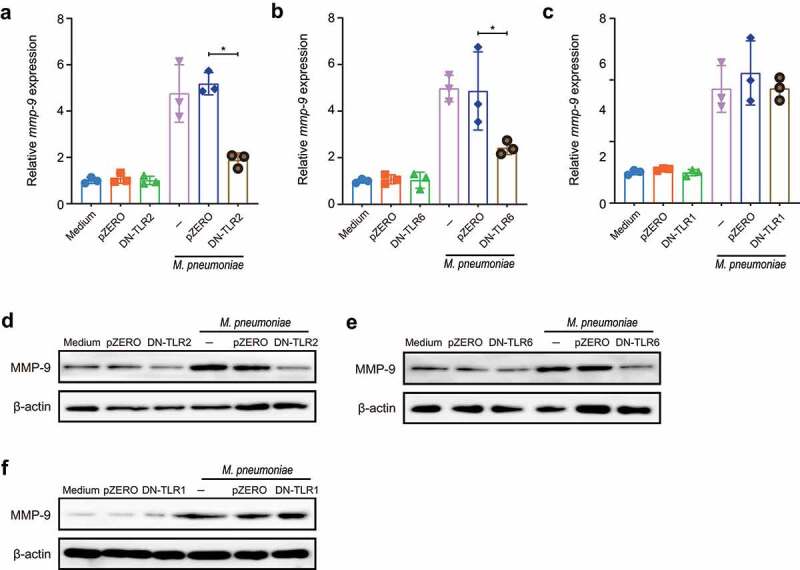
Inhibition of TLR2/6 shows reduced MMP-9 expression upon *M. pneumoniae* infection. BEAS-2B cells were plated in a 6-well plate, and TLR inhibition experiments were performed by transfection of 0.5 μg of empty vector (pZERO), DN-TLR1, DN-TLR2, or DN-TLR6 plasmid for 20 h before infection with *M. pneumoniae* (MOI 1:100) for another 16 or 24 h. Total RNA or protein was assayed for MMP-9 expression by qPCR (a–c) or immunoblotting (d–f). Experiments were repeated three times, and representative data are shown. *P < 0.05, compared with the indicated groups.

*M.pneumoniae*-triggered inflammatory responses are aided by the formation of TLR2/TLR1 or TLR2/TLR6 heterodimers [34]. To assess whether *M. pneumoniae*-induced MMP-9 expression was mediated through TLR1, TLR2, or TLR6, BEAS-2B cells were transfected with dominant negative (DN) plasmids of TLR1, TLR2, or TLR6 before infection with *M. pneumoniae* (Supplement materials Fig. S1). The data presented in Figure 2(a–b,d–e) revealed that both the mRNA and protein levels of MMP-9 were significantly inhibited by transfection of DN-TLR2 and DN-TLR6 plasmids. In contrast, the inhibition of TLR1 failed to abrogate *M. pneumoniae*-induced MMP-9 expression (Figure 2(c,f)), indicating that TLR2 and TLR6 receptors are involved in the induction of MMP-9. This hypothesis was further supported by the pretreatment with functional neutralizing antibodies targeting TLR1, TLR2, or TLR6 (Supplement materials Fig. S2). Moreover, the involvement of TLR2 and TLR6 in *M. pneumoniae*-induced MMP-9 expression was also verified in primary bronchial epithelial cells (Supplement materials Fig. S3). These results demonstrate that *M. pneumoniae*-induced MMP-9 expression in bronchial epithelial cells is mediated by TLR2 and TLR6.

### M. pneumoniae activates MAPKs to modulate MMP-9 expression

**Figure 3.**
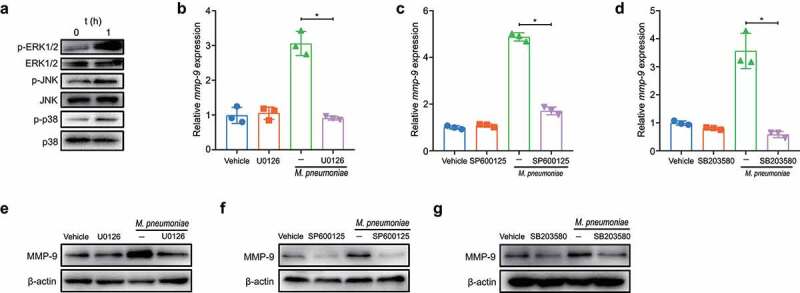
*M. pneumoniae* activates MAPKs to modulate MMP-9 expression. BEAS-2B cells were infected with 100 MOI of *M. pneumoniae* for 1 h and then phosphorylated, and total ERK1/2, JNK1/2, and p38 levels were detected by immunoblotting (a). For inhibitor experiments, cells were pretreated with 30 μM of MAPK inhibitors U0126 (ERK), SP600125 (JNK), or SB203580 (p38) for 30 min, and after 16 or 24 h of *M. pneumoniae* infection, MMP-9 mRNA (b–d) and protein (e–g) levels were detected by qPCR and immunoblotting, respectively. The experiment was repeated three times, and representative data are shown. *P < 0.05, compared with the control group.

The activation of TLR2 and TLR6 by *M. pneumoniae* is known to rapidly trigger the assembly of a supramolecular organizing centre (SMOC), known as the myddosome, to activate MAPKs in regulating inflammation [35–37]. We next investigated whether MAPKs participated in the regulation of MMP-9 expression following *M. pneumoniae* infection. The activation of MAPKs in BEAS-2B cells was initially validated by immunoblotting analysis, and the results showed that infection with *M. pneumoniae* for 1 h induced considerable phosphorylation of ERK1/2, JNK, and p38, while total kinase levels remained constant (Figure 3(a)). Pretreatment with the MAPK inhibitors U0126, SP600125, and SB203580 significantly reduced the transcription of MMP-9 mRNA (Figure 3(b–d)), and similar results were observed for protein levels in the immunoblotting analysis (Figure 3(e–g)). Our findings indicate that *M. pneumoniae*-stimulated MMP-9 expression is modulated by MAPKs in BEAS-2B cells.

### M. pneumoniae-stimulated MMP-9 expression requires MAPK/NF-κB

**Figure 4.**
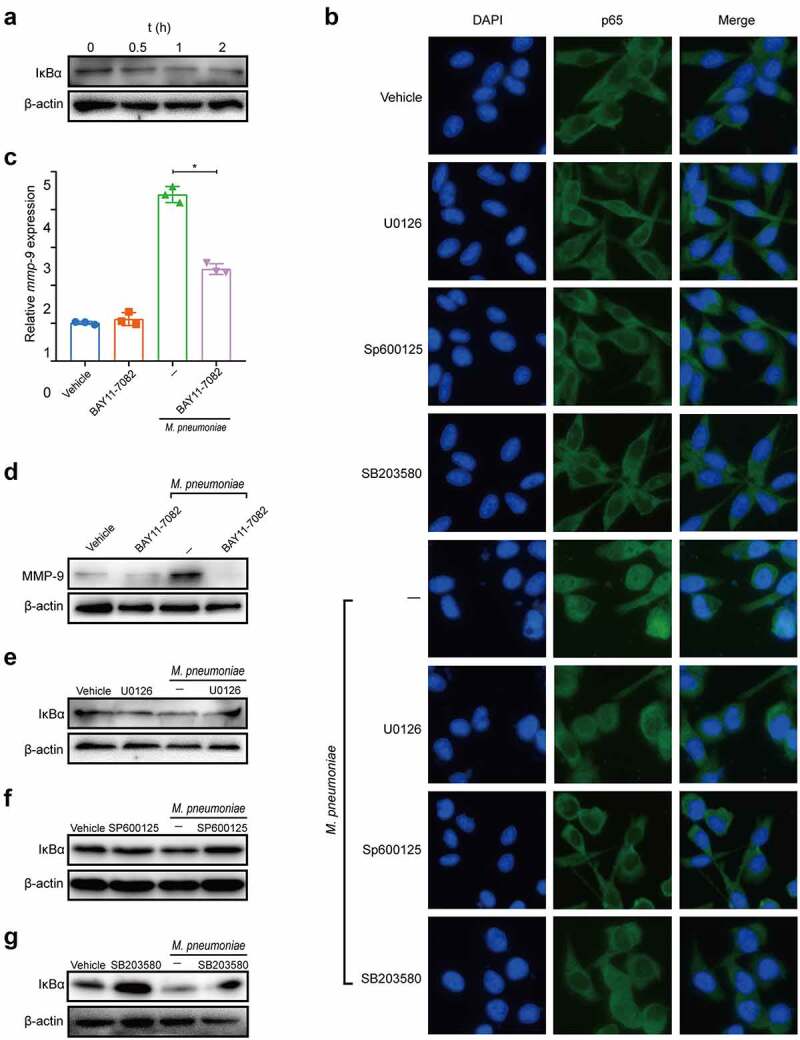
*M. pneumoniae*-stimulated MMP-9 expression requires MAPK/NF-κB. BEAS-2B cells were stimulated with 100 MOI of *M. pneumoniae* for 0, 0.5, 1, and 2 h. Then, IκBα expression was measured by immunoblotting (a). Cells were pretreated with or without MAPK inhibitors for 30 min before infection with *M. pneumoniae* for 1 h. Activation of NF-κB was assessed by immunofluorescence (b). BEAS-2B cells were preincubated with 5 μM BAY11–7082 for 30 min. After 16 h of infection, *MMP-9* mRNA expression was detected using qPCR (c,d). BEAS-2B cells were pretreated with MAPK inhibitors for 30 min before *M. pneumoniae* infection. IκBα expression was detected by immunoblotting (e–g). The results were obtained from three independent experiments performed in triplicate. *P < 0.05, compared with the indicated groups.

NF-κB modulates MMP-9 expression under various conditions [38], and NF-κB is activated by the JNK signalling cascade and related MAPKKK [39]. To investigate the role of NF-κB in *M. pneumoniae*-triggered MMP-9 expression and the relationship between MAPKs and the NF-κB signalling pathway, we detected changes in the inhibitory subunit of NF-κB and IκBα. As shown in Figure 4(a), IκBα was significantly degraded at 0.5–2 h after infection with *M. pneumoniae*. Immunofluorescence also revealed significant nuclear translocation of NF-κB 1 h post-infection (Figure 4(b)). As expected, the inhibition of NF-κB by BAY11–7082 significantly abrogated *M. pneumoniae*-induced MMP-9 expression (Figure 4(c,d)), suggesting that *M. pneumoniae*-induced MMP-9 expression is regulated by NF-κB. Furthermore, pretreatment with MAPK inhibitors reduced IκBα degradation induced by *M. pneumoniae* infection (Figure 4(e–g)) and inhibited the nuclear translocation of NF-κB (Figure 4(b)). These findings suggest that *M. pneumoniae*-induced MMP-9 expression depends on the MAPK/NF-κB signalling pathway.

### MMP-9 expression is upregulated by M. pneumoniae through AP-1

**Figure 5.**
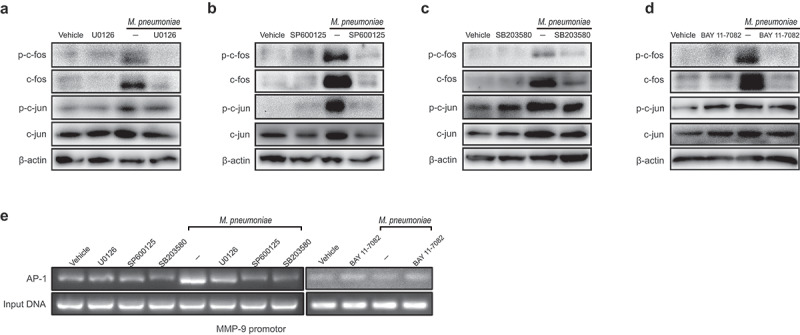
Requirement of AP-1 for efficient induction of MMP-9. Cells were seeded and infected with *M. pneumoniae* for 1 h. Phosphorylation of c-fos and c-jun was assessed by immunoblotting with or without specific inhibitors (MAPK inhibitors, 30μM and BAY11–7082, 5μM) (a–d). BEAS-2B cells were pre-incubated with or without inhibitors prior to *M. pneumoniae* infection. AP-1 recruitment to the *MMP-9* binding site was detected using a ChIP assay (e–f). Representative results from three independent experiments are shown.

The *MMP-9* promoter sequence contains activator protein-1 (AP-1) motifs to which members of c-fos and c-jun may bind [40]. To verify the involvement of AP-1 in the modulation of MMP-9 expression, we measured the phosphorylation levels of AP-1 subunits c-fos and c-jun following *M. pneumoniae* infection. The results clearly demonstrated that *M. pneumoniae* infection leads to AP-1 activation in BEAS-2B cells, and pretreatment with a MAPK or NF-κB inhibitor reversed these events (Figure 5(a–d)), indicating that *M. pneumoniae* activates AP-1 and that MAPK/NF-κB is the upstream signalling cascade for AP-1 activation. To further elucidate the effect of AP-1 on MMP-9 transcription, we conducted a ChIP assay to assess the binding of AP-1 to the predicted binding sites. As illustrated in Figure 4(e–f), exposure of the cells to *M. pneumoniae* promoted AP-1 binding to the *MMP-9* promoter. These findings supported the theory that the MAPK/NF-κB/AP-1 pathway is involved in MMP-9 expression during *M. pneumoniae* infections.

### Histone acetylation is required for M. pneumoniae-induced MMP-9 expression

**Figure 6.**
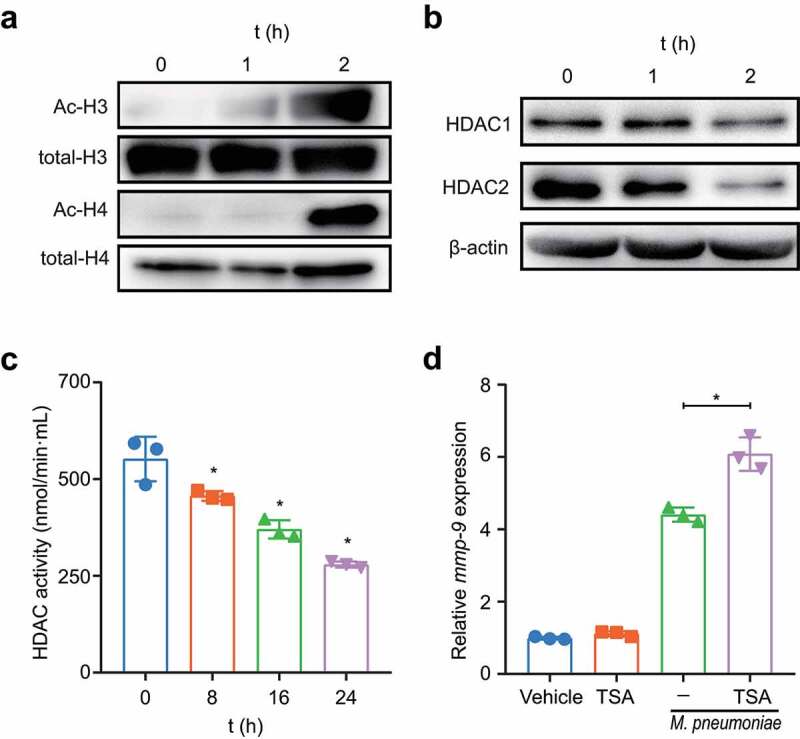
Histone acetylation is required for *M. pneumoniae*-induced MMP-9 expression. Cells were seeded and infected with *M. pneumoniae* (MOI = 100) for 0–2 h, and the acetylation status of histones H3 and H4, as well as the expression of HDAC1 and HDAC2 in cells, were detected by immunoblotting (a,b). (c) Total HDAC activity in *M. pneumoniae*-infected cells was measured (c). BEAS-2B cells were pretreated with an HDAC inhibitor (TSA, 100 ng/ml) for 30 min before infection with *M. pneumoniae* for 16 h, and qPCR was used to detect *MMP-9* mRNA expression (d). The results were obtained from three independent experiments performed in triplicate. *P < 0.05, compared with the indicated groups.

Histone acetylation was implicated in MMP-9 expression [41,42]. To confirm the role of this epigenetic modification in the modulation of MMP-9 expression following *M. pneumoniae* infection, we examined the acetylation status of histones H3 and H4 after *M. pneumoniae*-infection. Our findings indicated that *M. pneumoniae* infection led to a clear increase in the levels of acetylated H3 and H4 in BEAS-2B cells (Figure 6(a)). In general, increased histone acetylation is associated with decreased histone deacetylation, which is regulated by histone deacetylases (HDACs) [42]. Thus, we assessed the changes in the expression of HDAC1 and HDAC2 in *M. pneumoniae*-infected cells. As expected, we observed a significant decrease in the expression levels of both HDAC1 and HDAC2 following *M. pneumoniae* infection (Figure 6(b)). Additionally, the HDAC activity assay revealed a significant time-dependent decrease in intracellular HDAC activity compared with that in the uninfected control cells (Figure 6(c)). Furthermore, pretreatment with the HDAC inhibitor TSA resulted in increased MMP-9 transcription (Figure 6(d)). Taken together, these findings indicate that histone acetylation is required for *M. pneumoniae*-induced MMP-9 expression.

### M. pneumoniae infection increases the MMP-9/TIMP-1 ratio

**Figure 7.**
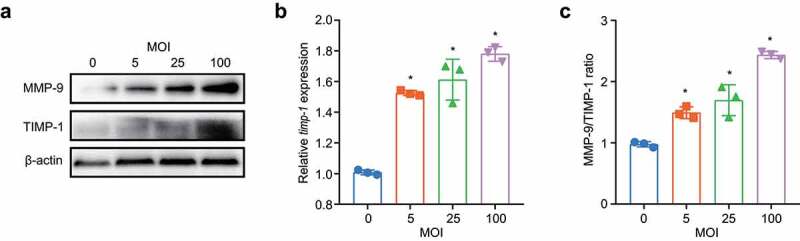
*M. pneumoniae* increases the MMP-9/TIMP-1 ratio. BEAS-2B cells were infected with *M. pneumoniae* (MOI = 0, 5, 25, and 100) for 16 or 24 h. TIMP-1 and MMP-9 expression was detected by qPCR and immunoblotting, and the MMP-9/TIMP-1 ratio was calculated (c). Representative results from three independent experiments are shown. *P < 0.05, compared with the control group (0 MOI).

TIMP-1 is presumed to be a natural inhibitor of MMP-9 [43,44]. To determine whether the increase in MMP-9 expression was associated with decreased TIMP-1 expression, we measured TIMP-1 expression in BEAS-2B cells after *M. pneumoniae* infection. Although both the mRNA and protein levels of TIMP-1 increased as the MOI increased (Figure 7(a,b)), the ratio of MMP-9/TIMP-1 increased in an MOI-dependent manner (Figure 7(c)).

### M. pneumoniae decreases RECK expression by activating Sp1 to promote MMP-9 activity

**Figure 8.**
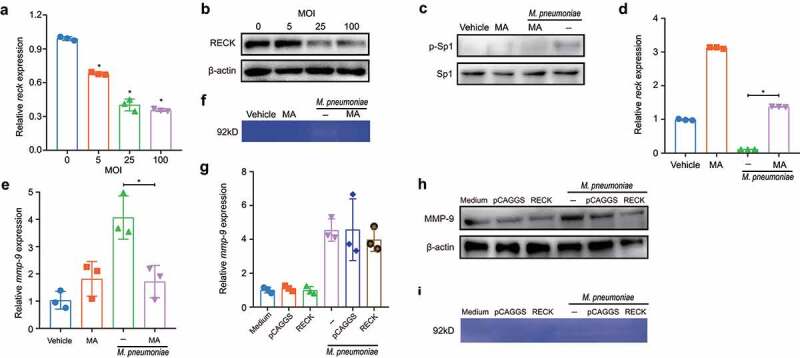
*M. pneumoniae* activates Sp1 to downregulate RECK expression. BEAS-2B cells were infected with different MOIs of *M. pneumoniae* for 16 h. RECK expression was measured using qPCR and immunoblotting (a,b), and the phosphorylation level of Sp1 was determined using immunoblotting (c). The cells were pretreated with an Sp1 inhibitor (mithramycin A; MA; 2 μM) for 30 min before being infected with *M. pneumoniae* for 16 h. The expression of *RECK* and *MMP-9* was assessed using qPCR (d,e), and MMP-9 enzyme activity was determined by gelatin zymography (f). BEAS-2B cells were transfected with a RECK-overexpressing plasmid and then infected with *M. pneumoniae* for 16 h. MMP-9 expression and enzymatic activity were measured by qPCR, immunoblotting, and gelatin zymography (g–i). Results are from three independent experiments performed in triplicate, *P < 0.05, compared with the control group (0 h) or the indicated group.

RECK is a recently discovered inhibitor of MMP-9 that blocks its secretion and catalytic activity [45]. To investigate the regulatory role of RECK in MMP-9 secretion, we evaluated the effect of *M. pneumoniae* infection on RECK expression. As depicted in Figure 8(a–b), both the mRNA and protein levels of RECK gradually decreased in *M. pneumoniae*-infected cells as the MOI increased. We also found that *M. pneumoniae* infection increased the phosphorylation level of nuclear transcription factor Sp1 in BEAS-2B cells (Figure 8(c)). Interestingly, pretreatment with the Sp1 inhibitor, mithramycin A (MA), abolished Sp1 phosphorylation and reversed the inhibitory effect of *M. pneumoniae* on both *RECK* mRNA expression (Figure 8(d)) and MMP-9 expression and activity (Figure 8(e–f)), indicating that *M. pneumoniae* decreased RECK expression via activation of Sp1. Additionally, overexpression of RECK in BEAS-2B cells had no significant impact on *M. pneumoniae*-induced MMP-9 transcription and translation (Figure 8(g–h)) but drastically decreased MMP-9 catalytic activity (Figure 8(i)), suggesting that RECK regulates MMP-9 activity in cells infected with *M. pneumoniae* infection.

## Discussion

Although monocytes and macrophages play dominant roles in the early immune response, only one alveolar macrophage patrol every three alveoli, implying that coordination with other cells is required to defend the large airway surface [46]. Airway epithelial cells are not just a simple layer of epithelial tissue; they share many characteristics with the immune cells. For example, these cells express pattern recognition receptors (PRRs), such as TLRs, NOD-like receptors, C-type lectins, and protease-activated receptors [47], which can recognize pathogen-associated molecular patterns (PAMPs) and lead to the reactive secretion of cytokines or chemokines, thereby facilitating the recruitment and activation of monocytes, macrophages, and dendritic cells (DCs) to amplify inflammatory responses [48]. In this study, we chose BEAS-2B cells to investigate *M. pneumoniae*-induced inflammation because this normal human bronchial epithelial cell line expresses multiple TLRs [49]. Furthermore, in contrast to other cancer-derived cells, the tumour suppressor RECK is expressed at normal levels in BEAS-2B cells [50]. After conducting an in-depth investigation of the mechanism by which *M. pneumoniae* induces an inflammatory response in bronchial epithelial cells, we obtained the following significant findings. First, *M. pneumoniae* infection was sufficient to induce the transcription, secretion, and enzymatic activity of MMP-9 in both BEAS-2B and primary bronchial epithelial cells. Second, the expression of MMP-9 was regulated by the TLRs/MAPK/NF-κB/AP-1 pathway, nuclear factor Sp1, and histone acetylation. Together, these factors formed a complex regulatory network that modulated MMP-9 expression. Finally, *M. pneumoniae* infection downregulated the expression of the tumour suppressor RECK, which is a natural inhibitor of MMP-9 secretion and activity. Low levels of RECK expression minimized the inhibitory effect of *M. pneumoniae* infection on MMP-9 release and enzymatic activity, thereby contributing to the production of active MMP-9 in the host. MMP-9 secretion during the acute phase of infection led to the degradation of ECM, which facilitates leukocyte transmigration from the vasculature to tissues. However, long-term or recurrent MMP-9 activation may result in airway injury or further aggravate airway remodelling in patients with COPD or asthma. Additionally, MMP-9 regulated the survival of inflammatory cells, as well as the availability and activity of inflammatory mediators. These factors contribute to the amplification of immune responses, which are critical for controlling infections, and provide an additional layer of control that helps fine-tune the inflammatory status.

Regulation of MMP-9 expression and secretion has been a hot topic [22]. In the present study, we demonstrated that TLR2 and TLR6 play crucial roles in the expression of MMP-9, which is consistent with the theory that the innate immune system recognizes *Mycoplasma* or diacylated lipoproteins by the formation of TLR2/TLR6 heterodimers [51]. TLR1 is involved in the activation of NF-κB by *M. pneumoniae*-derived lipoproteins (F0F1-ATPase, MPN611 and MPN162) [34,52]. However, our results showed that TLR1 seems somewhat dispensable in the induction of MMP-9; one plausible explanation may be the abundance these lipoproteins (i.e. F0F1-ATPase) didn’t reach the critical concentration to interact with TLR1, since the majority of the available lipoproteins were diacylated lipoproteins, which are recognized by TLR2 and TLR6 [53]. We also revealed that the MAPK signalling pathways, including ERK1/2, JNK1/2, and p38 are involved in the production of MMP-9. Activated MAPKs can stimulate downstream serine/threonine protein kinases or nuclear transcription factors with binding sites located in the promoter region (approximately 2.2 kbp in the 5′ region) of the *MMP-9* gene, such as NF-κB, AP-1, and Sp1 [54]. As postulated, our findings also showed that *M. pneumoniae* infection activates NF-κB and phosphorylates the AP-1 subunits c-fos and c-jun, both of which are involved in the regulation of MMP-9. Although AP-1 has been identified as the primary nuclear transcription factor regulating MMP-9 expression, it may not effectively induce MMP-9 expression. In many cases, NF-κB, Sp1, and other nuclear transcription factors act synergistically for AP-1 to function effectively [55]. Using specific inhibitors, we demonstrated that the MAPK/NF-κB/AP-1 signalling cascade was essential for MMP-9 production. These findings are in accordance with those of a previous study, which showed that increased MMP-9 expression induced by IL-33 was dependent on the NF-κB/AP-1 signalling pathway [56]. We also confirmed that Sp1 was involved in the induction of MMP-9 expression by *M. pneumoniae*. However, the mechanism by which TLR activation following *M. pneumoniae* infection contributes to Sp1 phosphorylation remains unclear, and further studies are needed to understand the precise regulatory mechanisms, as well as the crosstalk between MAPK/NF-κB/AP-1 and Sp1.

In eukaryotic cells, histone acetylation and deacetylation are maintained at dynamic equilibrium by histone acetyltransferases (HATs) and HDACs [57]. HDACs are a family of enzymes that deacetylate histones inside the chromatin structure and bind tightly to negatively charged DNA, resulting in densely coiled chromatin that inhibits gene transcription [58]. The significance of acetylation of histones H3 and H4 as well as the downregulation of class I HDACs (e.g. HDAC1 and HDAC2) in *M. pneumoniae*-stimulated MMP-9 expression was presented in the current study, and pretreatment with the broad-spectrum HDAC inhibitor TSA increased MMP-9 expression, indicating that *M. pneumoniae* can facilitate MMP-9 expression via acetylation of histones H3 and H4. However, the mechanism underlying the acetylation of H3 and H4 remains unclear. Our unpublished results indicated that *M. pneumoniae* causes metabolic reprogramming of airway epithelial cells, resulting in an altered tricarboxylic acid cycle and citrate build-up. During LPS-induced metabolic reprogramming, citrate is exported from the mitochondria to the cytosol and then metabolized to acetyl-coenzyme A (acetyl-CoA) by ATP citrate lyase (ACLY) [59]. Increased availability of acetyl-CoA eventually promotes the de novo acetylation of histones. Therefore, we cannot rule out the possibility that *M. pneumoniae*-induced histone acetylation was mediated by citrate-derived acetyl-CoA. Further studies are required to determine the precise mechanism of histone acetylation.

The most novel finding in our study was the implication of the tumour suppressor RECK in the inflammatory response induced by *M. pneumoniae*. Studies have shown that the K23 domain of RECK binds MMP-9 and inhibits its catalytic activity [60]. RECK expression is high in normal cells but is reduced or absent in malignant cells. Our findings indicated that RECK expression was downregulated by *M. pneumoniae* infection, which may account, at least in part, for the enhanced activity of MMP-9 following *M. pneumoniae* infection. This hypothesis was further supported by the observation that the enzymatic activity of MMP-9 was reduced following transfection with a RECK-overexpressing plasmid [23]. However, we did not observe any significant increase in mRNA or protein expression of MMP-9 in RECK-overexpressing cells, which is in agreement with Takahashi’s conclusions [23] but differed from the findings of previous studies by Takagi, who reported that MMP-9 transcription was suppressed in RECK-transfected human fibrosarcoma HT1080 cells [58]. One plausible explanation is that RECK is predominantly anchored on the cell membrane and does not directly enter the nucleus to bind to the promoter region of MMP-9, but may interact with other receptors on the plasma membrane to regulate MMP-9 transcription. However, this effect is negligible because it is insufficient to counteract *M. pneumoniae*-driven MMP-9 transcription. Nonetheless, the regulatory mechanisms underlying RECK expression in *M. pneumoniae* remain poorly understood. Lower RECK expression in cancer cells is frequently accompanied by epigenetic modifications, such as DNA methylation, and whether *M. pneumoniae* reduces RECK expression through methylation remains to be determined. Additionally, RECK expression has been shown to be regulated by microRNAs and the nuclear transcription factors NF-κB and Sp1 [60,61]. Sp1 is a member of the zinc finger family that binds specifically to GC-rich DNA sequences. Sp1 is overexpressed in most tumours and can inhibit RECK transcription by binding to its promoter region [59]. We demonstrated that the levels of phosphorylated Sp1 significantly increased following *M. pneumoniae* infection. Pharmacological inhibition of Sp1 reversed the downregulation of RECK expression induced by *M. pneumoniae*, as well as the transcription of MMP-9. This indicated that *M. pneumoniae* activated Sp1, which in turn inhibited RECK expression and thereby controlled MMP-9 activity.

**Figure 9.**
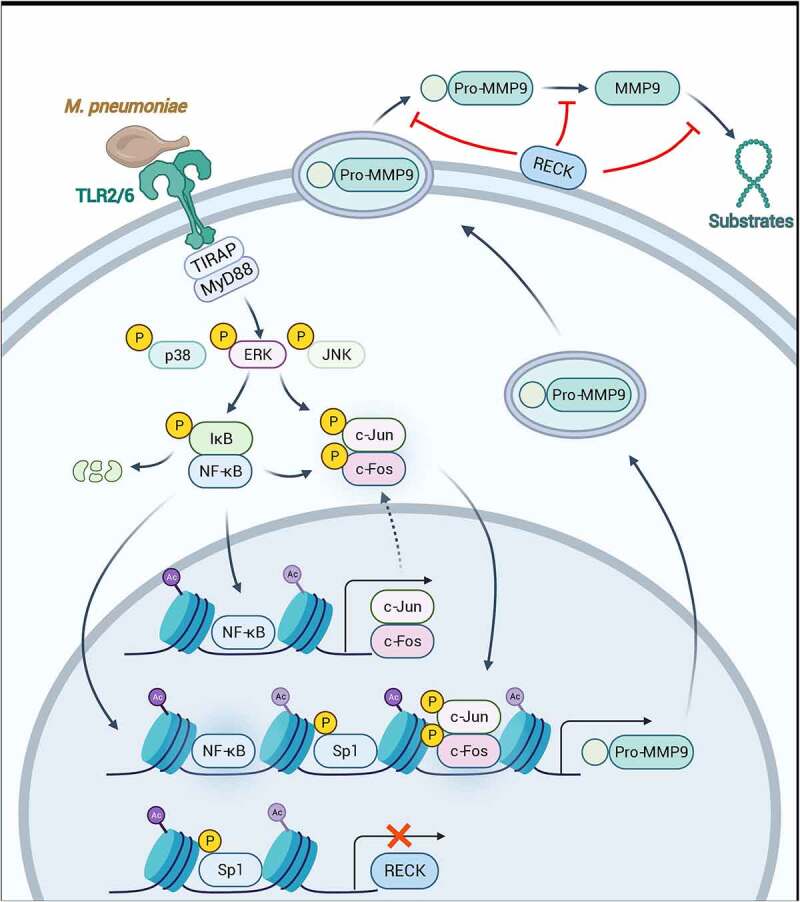
Schematic model depicting the potential signaling pathway involved in *M. pneumoniae*-induced MMP-9 secretion in bronchial epithelial cells. *M. pneumoniae* induces MMP-9 expression by activating the MAPK/NF-κB/AP-1 cascade, modulated by TLR2 and TLR6. *M. pneumoniae* induces the acetylation of histone and phosphorylation of Sp1 by unknown mechanisms. Histone acetylation facilitates MMP-9 expression, while Sp1 phosphorylation results in the downregulation of RECK. Decreased RECK expression weakened the inhibitory effect on MMP-9 release and enzymatic activity, thereby contributing to the secretion of active MMP-9 in the host (The figure is created with BioRender.Com).

In summary, our findings shed light on how *M. pneumoniae* stimulates MMP-9 expression in airway epithelial cells, thereby causing an inflammatory response (Figure 9). Our data support the following theories of MMP-9 secretion by *M. pneumoniae*: first, MMP-9 expression is associated with the MAPK/NF-κB/AP-1 cascade; second, histone acetylation appears to be critical in *M. pneumoniae*-induced inflammatory responses, indicating that epigenetic modifications induced by TLRs may be a notable inflammatory signal during bacterial infection. Our findings suggest that in addition to its function as a tumour suppressor, RECK may also play a role in infectious inflammation. Although there are far more questions than answers, elucidating the secondary roles of tumour suppressors could be beneficial for the development of novel therapeutic strategies for the management of inflammation.

## Supplementary Material

Supplemental Material

## Data Availability

The data supporting the findings of this study are available from Xiaoxing You upon reasonable request.
