# Supplementary material for: Mycoplasma pneumoniae downregulates RECK to promote matrix metalloproteinase-9 secretion by bronchial epithelial cells
Source: Virulence. 2022 Jul 26;13(1):1270–84. doi: 10.1080/21505594.2022.2101746 (PMC9336473; doi:10.1080/21505594.2022.2101746)
Supplement: Supplemental Material [file KVIR_A_2101746_SM2163.docx]

**Qin et al. Supplemental Materials**

**Materials**

pZERO-hTLR1, pZERO-hTLR2, and pZERO-hTLR6 dominant negative plasmids were obtained from InvivoGen (San Diego, CA), anti TLR2 IgA, anti TLR6 IgG and anti-TLR1 IgG neutralizing antibodies were purchased from InvivoGen (San Diego, CA).

**Supplementary figures**

**
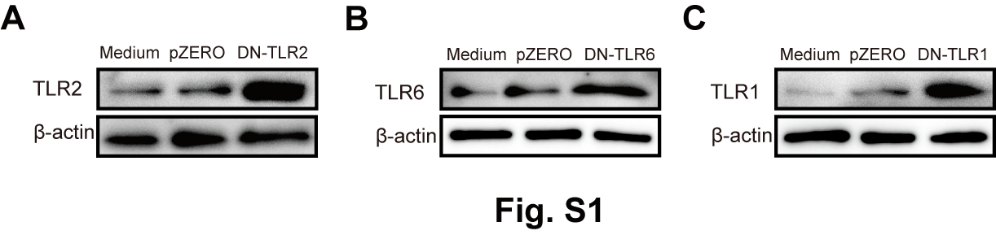
**

**Figure S1. Immunoblotting analysis for TLRs expression in BEAS-2B cells after dominant negative plasmid transfection.** BEAS-2B cells were cultured in 6-well plate at the concentration of 10^5^ for 24h, then cells were transiently transfected with a dominant negative (DN)-TLR2 (A), DN-TLR6 (B) or DN-TLR1 (C) plasmid. After 20 h of transfection, the cell lysates were prepared and separated by SDS-PAGE for immunoblotting analysis of TLR2, TLR6 or TLR1 expression by using the corresponding antibodies. Experiments were repeated three times, and representative data are shown.


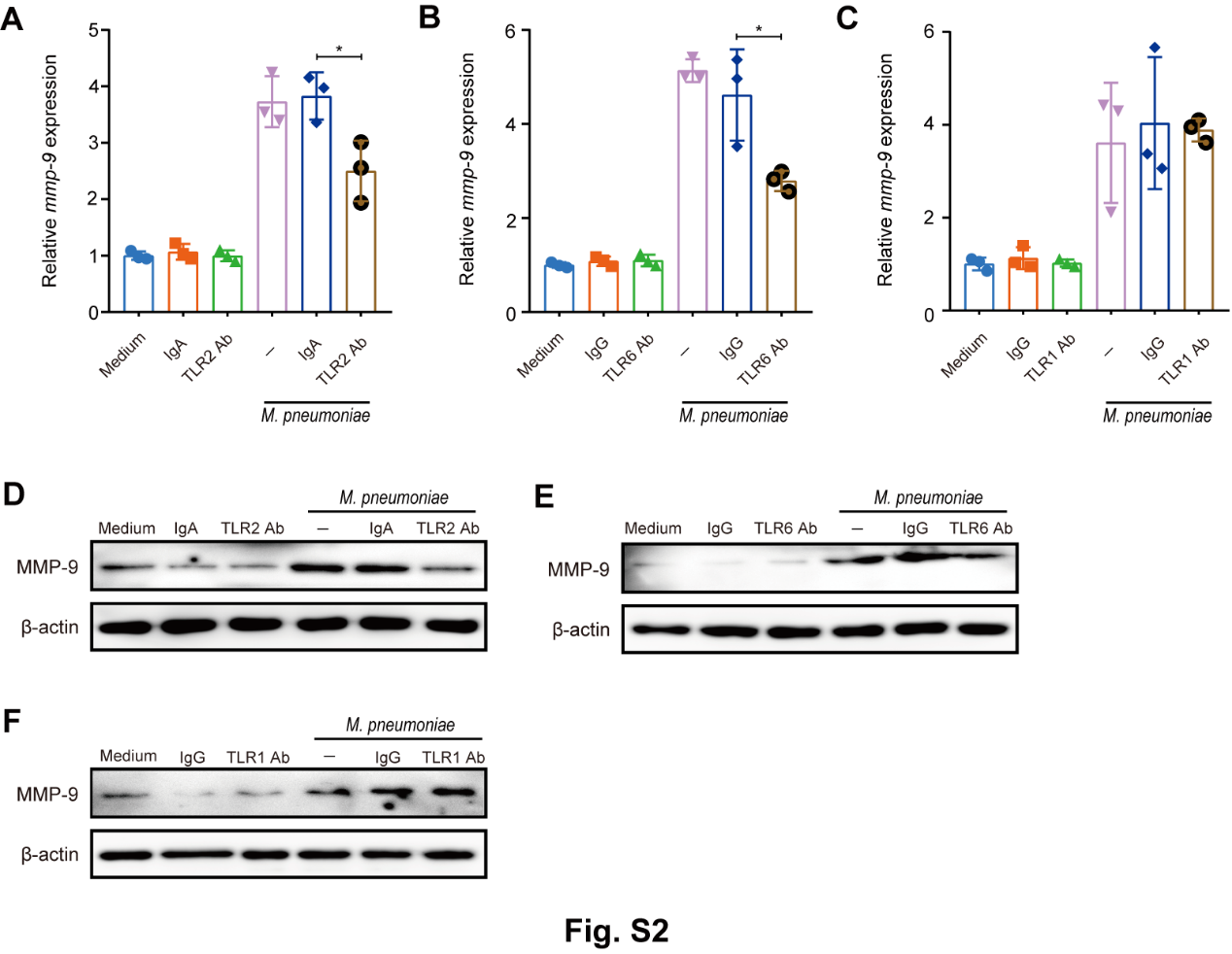


**Figure S2. Inhibition of TLR2/6 shows reduced MMP-9 expression upon *M. pneumoniae* infection.** BEAS-2B cells were preincubated with anti-TLR2 (10 μg/ml), anti-TLR6 (1 μg/ml), or anti-TLR1 (1 μg/ml) neutralizing antibodies for 1 h, and then infected with *M. pneumoniae* (MOI 1:100) for another 16 or 24 h. Total RNA or protein was assayed for MMP-9 expression by qPCR (A–C) or immunoblotting (D–F). Experiments were repeated three times, and representative data are shown. **P* < 0.05, compared with the indicated groups.

**
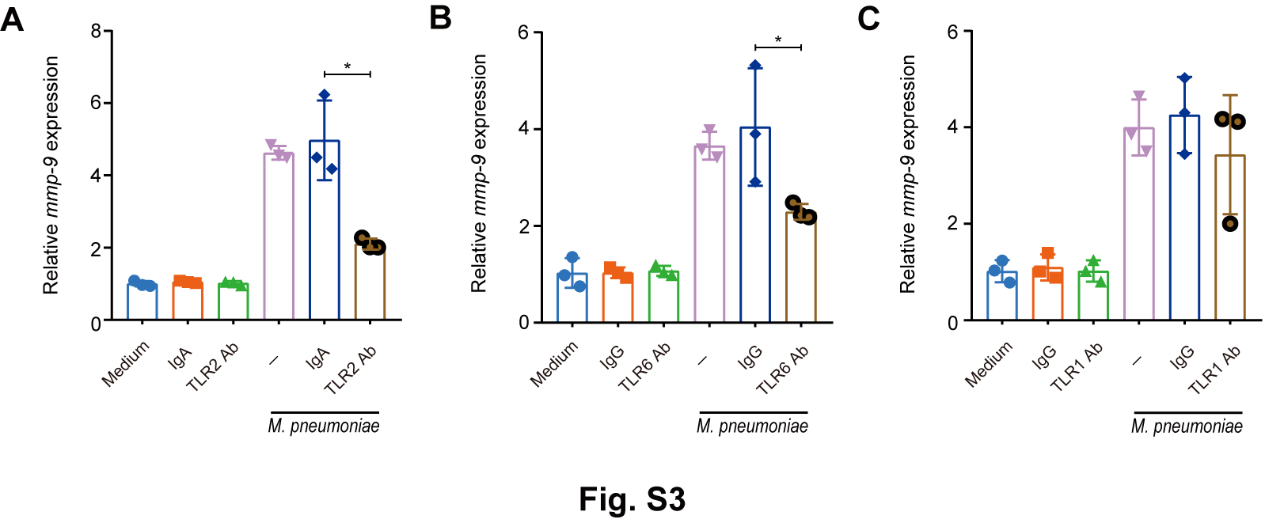
**

**Figure S3. *M. pneumoniae* infection induces MMP-9 expression in primary bronchial epithelial cells.** Primary bronchial epithelial cells were preincubated with anti-TLR2 (10 μg/ml), anti-TLR6 (1 μg/ml), or anti-TLR1 (1 μg/ml) neutralizing antibodies for 1 h, and then infected with *M. pneumoniae* (MOI 1:100) for another 16 or 24 h. Total RNA or protein was assayed for MMP-9 expression by qPCR (A–C). Experiments were repeated three times, and representative data are shown. **P* < 0.05, compared with the indicated groups.
